# Fibrinolytic potential as a risk factor for postpartum hemorrhage

**DOI:** 10.3389/fmed.2023.1208103

**Published:** 2023-09-08

**Authors:** Daniel Gruneberg, Paula Braun, Herbert Schöchl, Tereza Nachtigall-Schmitt, Maik von der Forst, Kevin Tourelle, Maximilian Dietrich, Markus Wallwiener, Stephanie Wallwiener, Markus A. Weigand, Herbert Fluhr, Julia Spratte, Stefan Hofer, Felix Carl Fabian Schmitt

**Affiliations:** ^1^Department of Anesthesiology, Heidelberg University Hospital, Heidelberg, Germany; ^2^Ludwig Boltzmann Institute for Traumatology, The Research Center in Cooperation with Allgemeine Unfallversicherungsanstalt, Vienna, Austria; ^3^Department of Obstetrics and Gynecology, University of Heidelberg, Heidelberg, Germany; ^4^Division of Obstetrics, Department of Obstetrics and Gynecology, Medical University of Graz, Graz, Austria; ^5^Department of Anesthesiology, Kaiserslautern Westpfalz Hospital, Kaiserslautern, Germany

**Keywords:** fibrinolytic potential, PPH, ClotPro, TPA-test, viscoelastic hemostatic assay, point-of-care, VET

## Abstract

**Background:**

Postpartum hemorrhage (PPH) is still the leading cause of maternal morbidity and mortality worldwide. While impaired fibrin polymerization plays a crucial role in the development and progress of PPH, recent approaches using viscoelastic measurements have failed to sensitively detect early changes in fibrinolysis in PPH. This study aimed to evaluate whether women experiencing PPH show alterations in POC-VET fibrinolytic potential during childbirth and whether fibrinolytic potential offers benefits in the prediction and treatment of PPH.

**Methods:**

Blood samples were collected at three different timepoints: T0 = hospital admission (19 h ± 18 h prepartum), T1 = 30–60 min after placental separation, and T2 = first day postpartum (19 h ± 6 h postpartum). In addition to standard laboratory tests, whole-blood impedance aggregometry (Multiplate) and viscoelastic testing (VET) were performed using the ClotPro system, which included the TPA-test lysis time, to assess the POC-VET fibrinolytic potential, and selected coagulation factors were measured. The results were correlated with blood loss and clinical outcome markers. Severe PPH was defined as a hemoglobin drop > 4g/dl and/or the occurrence of shock or the need for red blood cell transfusion.

**Results:**

Blood samples of 217 parturient women were analyzed between June 2020 and December 2020 at Heidelberg University Women's Hospital, and 206 measurements were eligible for the final analysis. Women experiencing severe PPH showed increased fibrinolytic potential already at the time of hospital admission. When compared to non-PPH, the difference persisted 30–60 min after placental separation. A higher fibrinolytic potential was accompanied by a greater drop in fibrinogen and higher d-dimer values after placental separation. While 70% of women experiencing severe PPH showed fibrinolytic potential, 54% of those without PPH showed increased fibrinolytic potential as well.

**Conclusion:**

We were able to show that antepartal and peripartal fibrinolytic potential was elevated in women experiencing severe PPH. However, several women showed high fibrinolytic potential but lacked clinical signs of PPH. The findings indicate that high fibrinolytic potential is a risk factor for the development of coagulopathy, but further conditions are required to cause PPH.

## 1. Introduction

Postpartum hemorrhage (PPH) is defined as excessive bleeding in the context of childbirth. Accounting for one death every 7 min, PPH is still the leading cause of maternal morbidity and mortality worldwide (1). Despite all the efforts to improve the prevention and treatment of PPH, healthcare providers are facing an increasing number of cases (2, 3). The reasons behind this are the growing incidences of high-risk pregnancies and atony-related PPH (2). Furthermore, little progress has been made in the early recognition of women at increased risk (4).

To facilitate prevention, efficient tools to identify women at risk are needed (4). Unfortunately, to this date, the ability to predict PPH remains low (5). Typically, an interplay of uterine atony, trauma, and placental abnormalities leads to bleeding (1). PPH further progresses if disturbances in hemostasis occur and aggravate bleeding, whereby the fibrinolytic system plays a crucial role (6). Roberts et al. showed that hyperfibrinolysis is common in PPH, and malfunctioning of this delicate system determines maternal mortality and morbidity (7). Despite the beneficial effects of tranexamic acid (TXA) in PPH (8–10), prophylactic use of antifibrinolytic agents during childbirth is still under debate (11). Thus, Arnolds et al. concluded in 2020 that further research is needed to determine optimal methods to assess fibrinolytic activity in PPH (12). Assessment of fibrinolytic potential might facilitate the identification of women at elevated risk for excessive bleeding antepartum and help in the timely recognition of coagulopathy and the progress of PPH.

**Figure 1:**
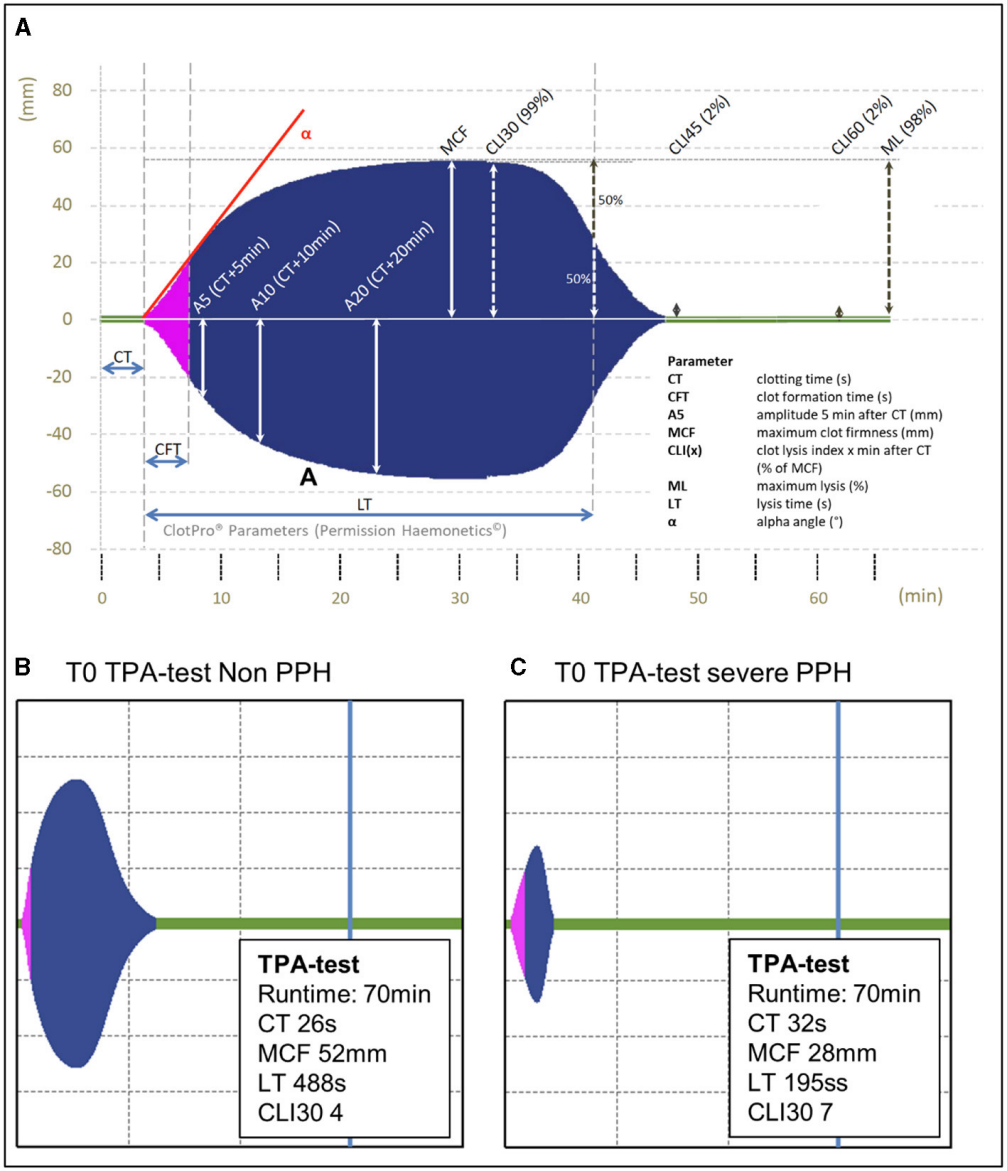
**(A)** ClotPro viscoelastic trace including the description of parameters. **(B, C)** Exemplary viscoelastic traces at T0 (prepartum) obtained from two women included in the study. **(B)** Shows the TPA-test trace of a woman with relatively low fibrinolytic potential and blood loss outside the normal range and **(C)** shows the TPA-test trace of a woman presenting with relatively high fibrinolytic potential at hospital admission and developing severe PPH in the course of delivery. ClotPro® hemostasis analyzer tracing image used by permission of Haemonetics Corporation. ClotPro® is a registered trademark of Haemonetics Corporation in the US, other countries or both.

The ClotPro TPA-test is a new viscoelastic point-of-care test assay to assess fibrinolytic potential. TPA-test stands for “tissue plasminogen activator-test.” The assay is, like the ex-test, a recombinant tissue factor-activated whole blood test. However, unlike the ex-test, the TPA-test contains a 650 ng/ml recombinant tissue plasminogen activator. The assay results in an extrinsically stimulated coagulation with r-tPA-stimulated hyperfibrinolysis. Typically, the viscoelastic trace shows the initiation of the clot comparable to the ex-test but is followed by fast lysis. Typical TPA-test viscoelastic traces are shown in Figures 1B, C. The fibrinolytic potential is measured as “lysis time,” which is defined as the timespan between clotting time and 50% clot lysis (Figure 1A). Typical TPA-test lysis time was reported to be ~220s in a patient population scheduled for elective orthopedic surgery (13). A higher fibrinolytic potential is expressed as shorter TPA-test lysis time (LT). To date, evidence of the clinical use of the ClotPro TPA-test is limited to a few reports. There are three retrospective analyses and one small case report showing prolonged TPA-test lysis time in patients with COVID-19 (14–17). One study and one case report utilized the TPA-test for detecting the TXA effect in cardiac surgery and elective orthopedic surgery (13, 18). Coupland et al. showed that the TPA-test LT indicates fibrinolytic resistance in critically ill patients, independent of COVID-19 (19). Finally, Nitsche et al. found in an experimental study that like other viscoelastic test parameters, TPA-test lysis time is temperature dependent and is prolonged in hypothermia (20).

To the best of our knowledge, fibrinolytic potential during pregnancy and childbirth has not yet been characterized.

To fill this knowledge gap, we evaluated whether women experiencing PPH show alterations in fibrinolytic potential during childbirth and whether ClotPro TPA-test lysis time may have a clinical value in the prediction and treatment of PPH. To answer these questions, blood samples of 217 women were taken at three different timepoints peripartum. TPA-test lysis time, laboratory coagulation status, clinical parameters, and short-term outcome were measured.

## 2. Materials and methods

### 2.1. Study design

The study was approved by the Ethics Committee of the Medical Faculty of Heidelberg (trial code no. S-759/2019) and is registered at the German clinical trials register (DRKS00021531). Data were collected prospectively in an observational single-center study at Heidelberg University Women's Hospital, a level-one perinatal center. Data were collected between June 2020 and December 2020.

Blood samples and clinical data of 217 women were collected peripartum. The inclusion criteria were age ≥18 years and written informed consent. The exclusion criteria were refusal of informed consent and age <18 years. To avoid potential bias, women with a medical history of coagulopathy were excluded from the final analysis. Medical history was obtained by the gynecologist and anesthetist. In addition, individuals were excluded from further analysis of the fibrinolytic potential after they received TXA. TXA leads to a strong inhibition of fibrinolysis, resulting in an artificial prolongation of TPA-test lysis time.

### 2.2. Study groups

As described in previous reports and international guidelines, mild PPH was defined as estimated blood loss >500 ml and hemoglobin drop >3 g/dl (21–23). Severe PPH was defined as the appearance of one of the following criteria: peripartum hemoglobin drop of ≥4 g/dl and transfusion of red blood cell concentrates or shock (24–29). Non-PPH was defined as a hemoglobin drop of ≤ 3 g/dl and estimated blood loss ≤ 500 ml (21–23).

### 2.3. Measurements

Blood samples were collected at three different timepoints. The first sample (T0) was taken at the time of hospital admission (19 h ± 18 h prepartum). The second (T1) was taken 30–60 min after placental separation and the third (T2) on the first day postpartum (19 h ± 6 h after placental separation). At each timepoint, blood samples were taken for viscoelastic testing (ClotPro), whole-blood impedance aggregometry (Multiplate), enzyme-linked immunosorbent assay (ELISA), and laboratory coagulation tests. All POC measurements were performed by the same experimenter. Citrate-anticoagulated whole blood was used for ClotPro analysis, and hirudin-anticoagulated whole blood was used for Multiplate analysis. All samples were transported from the delivery room or operation room to the research lab (5–10 min transportation time) at room temperature. Samples for POC VET and Multiplate were analyzed immediately in the research lab. Samples for routine coagulation testing were sent to the laboratory facility via tube mail and analyzed immediately after. Measurements of single coagulation factors were performed with frozen plasma samples. Therefore, blood samples were transported to the research lab, centrifuged at 3,000 rpm for 10 min, and plasma was aliquoted in 100 μl tubes. Samples were deep-frozen at −80°C and stored for 9 months. They were transported on dry ice to the laboratory facility for analysis and analyzed immediately after defreeze. At each timepoint, the following coagulation tests were performed for every patient.

### 2.4. Laboratory coagulation tests

Full blood count, hemoglobin, erythrocytes, hematocrit (Hct), prothrombin time, activated partial thromboplastin time, fibrinogen (Clauss fibrinogen assay, analyzed with the Sysmex CS 5100 coagulation analyzer), d-dimers, alpha-2-antiplasmin, factors V, VIII, IX, and XIII, protein C, protein S, von Willebrand factor (vWF) antigen, and activity were measured in the laboratory facility at Heidelberg University Clinic.

### 2.5. Point-of-care viscoelastic testing

For ClotPro (Enicor GmbH, Munich, Germany), we ran five channels for each blood sample (EX-test, IN-test, FIB-test, AP-test, and TPA-test). A detailed description of the TPA-test assay is provided in the introduction section of the present study. Based on the study by Tahitu et al. (30), the enzymatic fibrinolysis index was defined as Clotpro EX-test maximum clot lysis (ML) minus AP-test ML.

Whole-blood impedance aggregometry was performed with the Multiplate analyzer (Roche Diagnostics Deutschland GmbH, Mannheim, Germany). ADP-test, TRAP-test, and ASPI-test were performed for each sample at each timepoint.

All measurements were performed according to the manufacturer's guidelines.

### 2.6. Clinical data

Clinical data and patient characteristics were obtained from electronic medical reports. We documented maternal age, date and time of birth, gestational age, birth weight, gravida and parity, history of miscarriage, gemini status, mode of delivery, presence of placental pathologies, presence and degree of birth injury, presence of preeclampsia or HELLP syndrome, occurrence for postpartum uterine atony, clinical outcome (death, shock, hospital and intensive-care-unit length of stay, and need for re-operation), transfusion of red blood cell concentrate or fresh-frozen plasma, any medication given in the peripartum period (including uterotonics, TXA, fibrinogen concentrate, and vasopressors), and prescription of coagulant drugs). Estimated blood loss was documented according to the anesthesia protocol and/or birth report by midwives. The common causes of PPH were assessed according to the Four Ts mnemonic device for PPH (31).

### 2.7. Statistical analysis

Statistical analyses were performed using SAS version 9.4 (SAS Institute Inc., Cary, North Carolina, USA). For continuous data, ANOVA was performed for equal variances and a mixed linear model with Tukey–Kramer *post-hoc* test for unequal variances. A chi-squared test was performed for categorical data. For multiple comparisons of categorical data, *p*-value correction was made by performing a Bonferroni post-test. For ROC analysis, simple and multiple linear regression models were used. Probability values of *p* < 0.05 were generally considered statistically significant.

## 3. Results

### 3.1. Study groups

**Figure 2:**
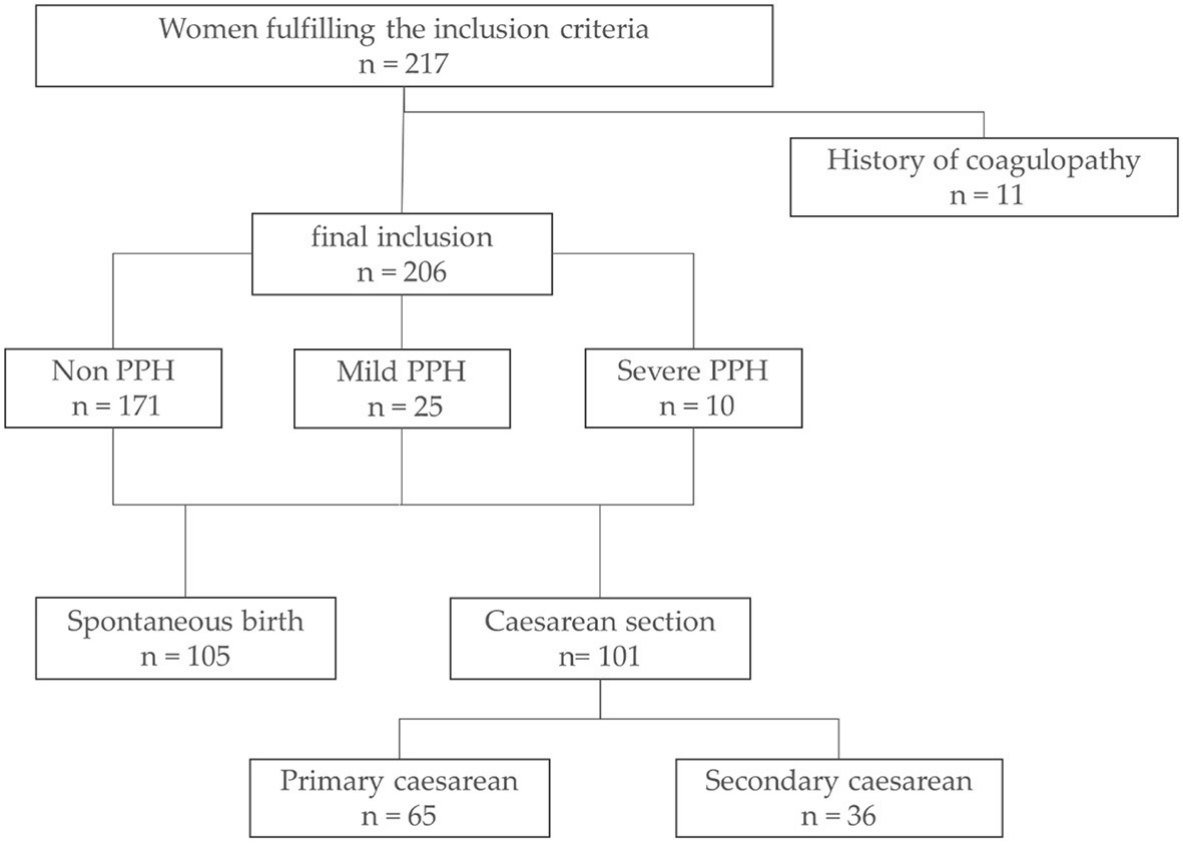
Data collection and inclusion. Flowchart of the data collection process and data exclusion. Definition of mild and severe PPH according to estimated blood loss, ΔHb, and shock, as described above. PPH, postpartum hemorrhage.

From 217 women initially recruited, 206 participants were eligible for final analysis (Figure 2). Eleven women had a medical history of coagulopathy (factor V Leiden mutation, von Willebrand disease, F XII deficiency, MTHFR mutation, and autoimmune thrombocytopenia either alone or in combination) and were subsequently excluded from further analysis. Three of 11 women with preexisting coagulopathy had a prescription for coagulation modifiers, which potentially influence coagulation and fibrinolysis. Patients were grouped based on the presence and severity of PPH: 25 (12.1%) women showed mild and 10 (4.9%) women showed severe PPH according to the aforementioned criteria of estimated blood loss, hemoglobin drop, red blood cell transfusion, and the presence of shock. The remaining 171 women revealed no evidence for PPH with estimated blood loss ≤ 500 ml and ΔHb ≤ 3g/dl. The mode of delivery did not differ significantly between groups.

### 3.2. Baseline characteristics

Before POC-VET fibrinolytic potential was evaluated, baseline characteristics, including sociodemographic data, parameters of pregnancy, and mode of delivery, were obtained. The data are outlined in Table 1.

**Table 1:** Demographic data and baseline characteristics based on the PPH group.

	**Total *n* = 206**	**Non-PPH *n* = 171**	**Mild PPH *n* = 25**	**Severe PPH *n* = 10**	***p*-value non vs. mild**	***p*-value non vs. severe**	***p*-value mild vs. severe**
Age (year)	33.4 ± 5.0	33.2 ± 4.9	33.4 ± 4.8	36.5 ± 5.7	0.80	**0.04**	0.10
Gestation age (week)	38.9 ± 1.4	38.9 ± 1.4	38.8 ± 1.9	39.1 ± 1.5	0.85	0.57	0.55
Obesity	53 (25.7%)	43 (25.2%)	7 (28.0%)	3 (30.0%)	0.76	0.73	0.91
Gravidity	2 (1–3)	2 (1–3)	2 (1–3)	2 (1–3)	0.42	0.86	0.54
Parity	1 (0–1)	1 (0–1)	0 (0–1)	0 (0–1)	0.65	0.82	0.95
Nullipara	97 (47.1%)	76 (44.4%)	14 (56.0%)	7 (70.0%)	0.28	0.11	0.45
Gemini	10 (4.9%)	6 (3.5%)	2 (8.0%)	2 (20.0%)	0.29	**0.01**	0.31
Preterm birth	14 (6.8%)	9 (5.3%)	4 (16.0%)	1 (10%)	**0.04**	0.52	0.65
Birthweight (g)	3,322 ± 502	3,307 ± 501	3,446 ± 467	3,268 ± 611.3	0.20	0.81	0.34
Spontaneous birth (*n*)	105	90 (53%)	8 (32%)	7 (70%)	0.59	0.48	0.17
Primary cesarian (*n*)	65	53 (31%)	11 (44%)	1 (10%)	0.16	0.85	0.12
Secondary cesarian (*n*)	36	28 (16%)	6 (24%)	2 (20%)	1.0	1.0	1.0

Data are presented as mean ± SD, median and IQR, or absolute and relative counts as appropriate; obesity was defined as BMI > 35, and preterm birth was defined as birth before 37+0 weeks of pregnancy.

PPH, postpartum hemorrhage.

The bold values indicate significant values of *p* < 0.05.

Women in the severe PPH group were older, with a mean difference of 3 years, and the proportion of gemini pregnancies was higher in this group than in the non-PPH group. The frequency of preterm birth was higher in the mild PPH group than in the non-PPH group. Other baseline characteristics were similar between the groups.

### 3.3. Follow-up

In the follow-up, maternal outcomes were negatively influenced by the severity of PPH. Increased blood loss in severe PPH was accompanied by a higher rate of shock, red blood cell transfusion, and the need for revision surgery. Women in the severe PPH group had a longer hospital length of stay and a higher ICU admission rate than those in the non-PPH and mild PPH groups. Data are presented in Supplementary Table 1.

At the time of T1 blood sampling, none of the parturient women presented with ongoing bleeding. One woman developed bleeding after T1 blood sampling. In all other cases, the bleeding had already stopped at timepoint T1.

### 3.4. Antepartum coagulation status

To determine antepartum differences in fibrinolytic potential, TPA-test lysis time and other indicators of clot initiation, firmness, and clot lysis were measured at the time of inclusion (T0).

Antepartum TPA-test lysis time was lower in the severe PPH group than in the non-PPH and mild PPH groups. Statistical significance was reached only in the comparison between the non-PPH and severe PPH groups (mean difference 21.7s; 95% CI −3.2 to 40.4; *p* = 0.04; Table 2). Shortened TPA-test lysis time indicates an increased fibrinolytic potential. However, 93 women (45% of the entire study population) showed low TPA-test lysis time <250 s but blood loss within the normal range of delivery. The proportion of low TPA-test lysis time in the non-PPH group was 54%, which increased to 70% in the severe PPH group. Women with low TPA-test lysis time and severe PPH showed significantly more risk factors for bleeding, according to the Four Ts mnemonic device (0.4 in the non-PPH vs. 1.0 in the severe PPH group, *p* = 0.005). Antepartum hemoglobin, Hct, and quantitative and qualitative platelet analyses, as well as classical POC-VET lysis parameters, measured as EX-test lysis index 30 and 60 did not differ between the groups and were within the normal range. In-test clotting time was prolonged in the mild PPH group, but the values were still within the normal range defined for pregnancy.

**Table 2:** Measurements of coagulation parameters antepartum (T0).

	**Non-PPH *n* = 171**	**Mild PPH *n* = 25**	**Severe PPH *n* = 10**	***p*-value non vs. mild**	***p*-value non vs. severe**	***p*-value mild vs. severe**
**Red blood cells**						
Hb (g/dl)	12.2 ± 1.0	12.4 ± 0.94	12.7 ± 1.2	0.40	0.13	0.39
Hct (%)	0.36 ± 0.03	0.36 ± 0.03	0.37 ± 0.04	0.56	0.13	0.32
**Clot initiation**						
CT Ex-test (s)	44.0 ± 7.6	46.6 ± 8.13	47.1 ± 8.6	0.11	0.21	0.86
CT In-test (s)	140.8 ± 10.4	145.44 ± 12.44	141.5 ± 11	0.04	0.83	0.32
**Clot firmness**						
Ex-test A10 (mm)	59.6 ± 3.8	60.4 ± 3.69	59.8 ± 3.9	0.31	0.85	0.67
Fib test A10 (mm)	24.2 ± 3.4	25.2 ± 3.93	25.1 ± 2.8	0.19	0.44	0.94
**Clot lysis**						
Ex-test CLI30	99.3 ± 0.8	99.16 ± 0.69	99.1 ± 0.6	0.55	0.53	0.83
Ex-test CLI60	94.4 ± 2.3	94.56 ± 2.47	94.9 ± 2.1	0.79	0.54	0.70
Ex-test ML	6.4 ± 2.6	6.5 ± 2.4	5.8 ± 2.2	0.80	0.49	0.45
AP test ML	6.1 ± 2.4	5.5 ± 2.0	5.0 ± 2.3	0.26	0.16	0.55
**Platelets**						
Platelet count (nl^−1^)	224.1 ± 63.7	217.2 ± 55.19	222.2 ± 64.1	0.61	0.93	0.83
TRAP AUC	103.7 ± 27.8	105.1 ± 29.5	105.1 ± 28.7	0.81	0.88	1.0
ASPI AUC	96.1 ± 27.3	95.32 ± 23.7	94.9 ± 21.2	0.89	0.89	0.97
ADP AUC	69.5 ± 24.9	71.4 ± 30.23	74.1 ± 24.9	0.74	0.58	0.76
**Fibrinolytic potential**						
TPA-test lysis time (s)	253.6 ± 50.4	258.5 ± 46.7	231.9 ± 24	0.89	**0.04**	0.11
D-dimers (mg/L)	2.2 ± 1.7	2.6 ± 2.3	3.0 ± 1.8	0.28	0.14	0.52

Measurements of coagulation parameters antepartum (T0). Data are presented as mean ± SD.

Hb, hemoglobin; Hct, hematocrit; CT, clotting time; A10, clot firmness after 10 min runtime; CLI30, clot lysis index after 30 min runtime; CLI60, clot lysis index after 60 min runtime; AUC, area under the curve; P1, non-PPH vs. mild PPH; P2, non-PPH vs. severe PPH; P3, mild PPH vs. severe PPH.

The bold values indicate significant values of *p* < 0.05.

### 3.5. Evaluation of the fibrinolytic system over the course of delivery

**Figure 3:**
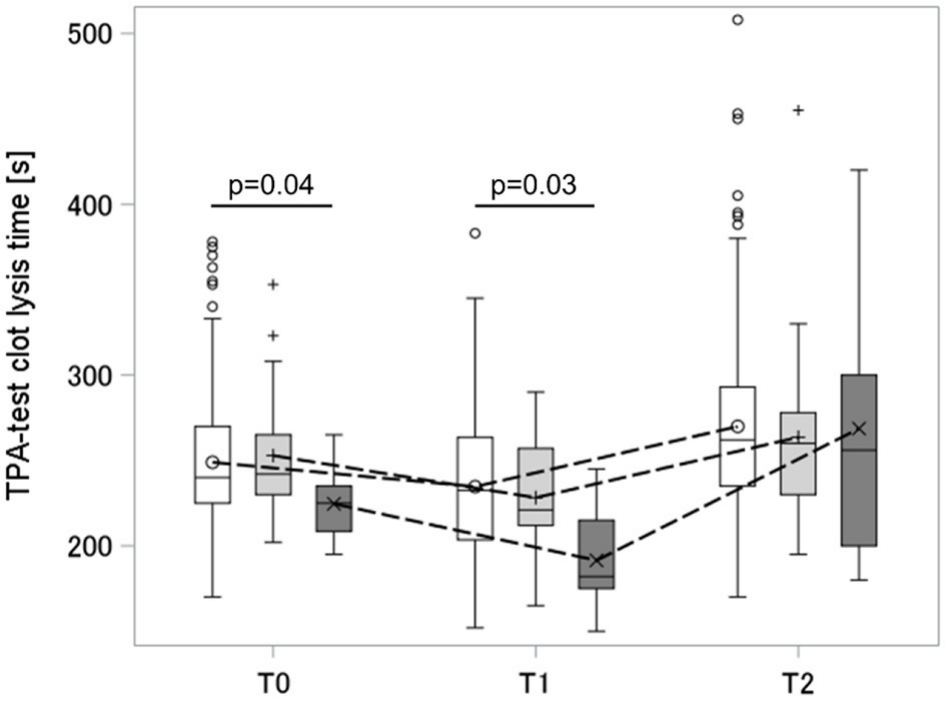
Dynamic of TPA-test lysis time peripartum. Box and whisker plot: The box indicates the Q1–Q3 inter-quartile range, the horizontal line inside the box shows the median value, the marker inside the box displays the mean, the whiskers indicate observation nearest to the fences (= 1.5 IQR), the markers below and above the fences demonstrate observations above and below the fences; T0, antepartum; T1, 30–60 min after placental separation; T2, first day postdelivery; groups: white, non-PPH; light gray, mild PPH; dark gray, severe PPH. PPH, postpartum hemorrhage.

In the following section, POC-VET fibrinolytic potential characterized over the course of delivery has been described. Figure 3 shows the TPA-test lysis time at three different timepoints during birth. At the time of hospital admission and 30–60 min after placental separation, the TPA-test lysis time was significantly reduced in the severe PPH group compared to the non-PPH group. On the first day postpartum, the TPA-test lysis time increased and exceeded the antepartum values, independent of the presence of PPH. Hence, there were no significant differences in fibrinolytic potential on this day.

Three individuals in the mild PPH group and two individuals in the severe PPH group received TXA over the course of delivery. While both women in the severe PPH group received TXA after T1 blood sampling, the three women in the mild PPH group received TXA between T0 and T1. After receiving TXA, the TPA-test lysis time values of these individuals were excluded from further analysis.

**Figure 4:**
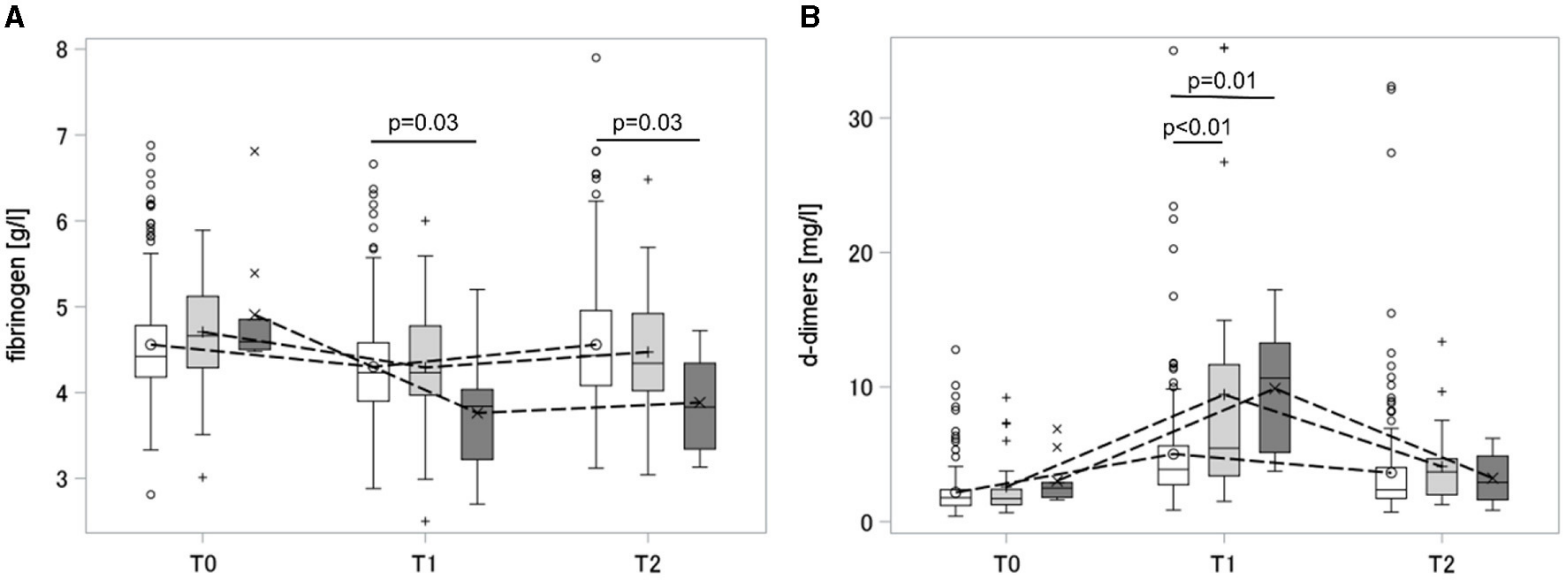
Dynamic of fibrinogen **(A)** and d-dimers **(B)** peripartum. Box and whisker plot: The box indicates the Q1–Q3 inter-quartile range, the horizontal line inside the box shows the median value, the marker inside the box displays the mean, the whiskers indicate observation nearest to the fences (=1.5 IQR), the markers below and above the fences demonstrate observations above and below the fences; T0, antepartum; T1, 30–60 min after placental separation; T2, first day postdelivery; groups: white, non-PPH; light gray, mild PPH; dark gray, severe PPH. PPH, postpartum hemorrhage.

As shown in Figure 4, increased fibrinolytic potential in severe PPH was accompanied by a significant drop in fibrinogen concentration and elevation in d-dimer levels 30–60 min after placental separation. Fibrinogen levels remained low on the first day postpartum. D-dimer-fibrinogen ratios were calculated for every timepoint and are shown in Supplementary Figure 1.

While the main inhibitors of the fibrinolytic system [alpha-2-antiplasmin and plasminogen activator inhibitor 1 (PAI-1)] did not differ antepartum (at the time when the fibrinolytic potential was already altered), they were reduced after placental separation, indicating a disinhibition of fibrinolysis. The data are shown in Table 3. PAI-1 drop after placental separation was more pronounced in the severe PPH group than in the mild PPH group. Overall, a greater extent of fibrinolysis disinhibition 30–60 min after placental separation was observed in the severe PPH group than in the other two groups. Correlations of TPA-test lysis time with fibrinogen, d-dimer, alpha-2-antiplasmin, and PAI-1 levels were calculated as measures of increased fibrinolysis. TPA-test lysis time showed low to moderate correlation with fibrinogen, d-dimer, alpha-2-antiplasmin, and PAI-1 levels. The data are shown in Supplementary Table 2.

**Table 3:** Measurements of fibrinolytic inhibitors over time.

	**Non-PPH *n* = 171**	**Mild PPH *n* = 25**	**Severe PPH *n* = 10**	***p*-value non vs. mild**	***p*-value non vs. severe**	***p*-value mild vs. severe**
**Alpha-2-antiplasmin**						
T0	96.7 ± 18.5	95 ± 12.6	87.7 ± 33.3	0.68	0.14	0.30
T1	87.4 ± 20	86.6 ± 16.1	68.3 ± 17	0.85	**< 0.01**	**0.02**
T2	98 ± 17.9	93.4 ± 11.8	82.1 ± 11.4	0.21	**0.01**	0.11
**PAI-1**						
T0	67.1 ± 28.6	68.8 ± 33.2	74.7 ± 48.3	0.79	0.44	0.60
T1	42.7 ± 23.3	58.2 ± 55.6	41.7 ± 42	**0.02**	0.93	0.18
T2	26.8 ± 14.1	24.1 ± 13.7	72.7 ± 85	0.53	**< 0.01**	**< 0.01**

PAI-1, plasminogen activator inhibitor 1; T0, at hospital admission; T1, 30–60 min after placental separation; T2, first day postpartum; P1, non-PPH vs. mild PPH; P2, non-PPH vs. severe PPH; P3, mild PPH vs. severe PPH.

The bold values indicate significant values of *p* < 0.05.

Parameters with potential impact on the hemostatic process, including Hct, Hb, thrombocyte counts, vWF, and factor XIII were measured at all timepoints, and the data are given in Supplementary Table 4. In summary, Hb and Hct were significantly decreased at timepoints T1 and T2 in the severe PPH group compared with the non-PPH group, while thrombocyte counts remained stable. vWF activity was increased at hospital admission in the severe PPH group (Supplementary Table 4).

Finally, TPA-test lysis time was compared to classic POC-VET fibrinolysis parameters. Enzymatic fibrinolytic index (EFI) was calculated as a marker of fibrinolysis insensitive to clot retraction. None of the 206 individuals in this study reached the common cut-off of EFI >15% for hyperfibrinolysis.

### 3.6. Incidence of additional risk factors for PPH

It was observed that insufficient uterine tone, placental retention, and birth injuries were more common in the severe PPH group. The data are presented in Table 4.

**Table 4:** Risk factors for the development of PPH and the use of uterotonics.

	**Non-PPH *n* = 171**	**Mild PPH *n* = 25**	**Severe PPH *n* = 10**
**Tone**			
Insufficient uterine contraction *n* (%)	0 (0%)	2 (8.0%)	2 (20.0%)
Oxytocin usage *n* (%)	143 (83.6 %)	24 (96.0%)	9 (90.0%)
Sulprostone usage *n* (%)	0	1 (4%)	0
**Tissue**			
Retention of placenta *n* (%)	0	0	3 (30.0%)
Placenta previa	1 (0.5%)	2 (8%)	0
PAS disorder *n* (%)	2 (1.1%)	0	0
**Trauma**			
Birth injury *n* (%)	68 (39.8%)	8 (32.0%)	7 (70.0%)

Additional risk factors for PPH according to the Four Ts mnemonic device. Values are extracted from electronic medical reports and presented as absolute counts and relative proportions. Insufficient uterine contraction clinically diagnosed by obstetricians.

PAS disorder, placenta accrete spectrum disorder.

To evaluate the role of uterine atony as a potential confounder of the influence of fibrinolytic potential on bleeding severity, we performed a subgroup analysis of fibrinolytic potential for women with and without uterine atony. Since only two individuals had documented uterine atony in the PPH groups, it was not possible to calculate meaningful statistics. However, TPA-test lysis time was shorter in the severe PPH group than in the non-PPH and mild PPH groups at timepoints T0 and T1, both in the subset with and without uterine atony. The data are presented in Supplementary Table 3.

### 3.7. ROC analysis–clinical value of antepartum TPA-test lysis time in the prediction of PPH

ROC analysis revealed a low predictive value of antepartum TPA-test lysis time as a single parameter. For the prediction of severe PPH, AUC was 0.634 (*p* = 0.053). When combined with fibrinogen, the predictive power increased (AUC 0.732; *p* = 0.002). The best prediction of severe PPH was achieved in a multiple linear regression model using TPA-test lysis time and the number of risk factors, according to the 4Ts (AUC 0.75; *p* = 0.0003).

## 4. Discussion

In the current study, we evaluated alterations of the POC-VET fibrinolytic potential in PPH. We demonstrated that women who developed severe PPH in the course of delivery show increased fibrinolytic potential already at the time of hospital admission. We also demonstrated that the difference in fibrinolytic potential persists 30–60 min after placental separation. Furthermore, we revealed that childbirth is accompanied by changes in fibrinolytic potential, independent of the occurrence of PPH. Parturient women showed an increase in fibrinolytic potential within the first hour after placental separation, followed by a decrease that exceeded the baseline level on the first day postdelivery.

The fact that fibrinolytic potential in the severe PPH group was already increased at the time of hospital admission indicates that the ClotPro TPA-test lysis time could identify altered fibrinolysis earlier than classical POC-VET fibrinolysis markers. This offers potential clinical use in the prevention and timely treatment of PPH. The fact that TPA-test lysis time indeed measures amplified fibrinolysis is supported by correlations between increased fibrinolytic potential, disinhibition of fibrinolysis (reduced PAI-1 and alpha-2-antiplasmin), and fibrinogen consumption markers (drop in fibrinogen and rise in d-dimers).

ROC analysis revealed that TPA-test lysis time alone is not specific enough to predict PPH. However, a combination of antepartum TPA-test lysis time with either fibrinogen levels or the number of risk factors according to the 4Ts improves the prediction of severe PPH. Nevertheless, we found important restrictions in the clinical applicability of TPA-test lysis time. A high fibrinolytic potential was found in 54% of individuals in the non-PPH group, leading to an AUC of only 0.6434 for TPA-test lysis time to predict severe PPH. Women presenting with high fibrinolytic potential but without PPH showed significantly fewer risk factors according to the Four Ts mnemonic device for PPH than women with severe PPH. Our interpretation of the results is that increased fibrinolytic potential should be understood as a moderating variable that increases the risk of developing coagulopathy, while high fibrinolytic potential alone is not sufficient for causing excessive bleeding. Additional factors such as uterine atony, soft tissue trauma, and placental pathologies are needed to cause severe PPH.

While our data provide new insights into the measurement of POC-VET fibrinolytic potential during childbirth, our study also has some limitations. First, blood loss was estimated by the obstetric team. However, no exact blood loss measurements were performed using calibrated blood bags because such systems were not established at the site of investigation, and implementation in a multidisciplinary setting would have surpassed the capabilities of this study. Since technical aids for blood loss measurement are not established to a large extent, hemoglobin and hematocrit are established as common parameters to objectify blood loss in clinical studies (24, 26, 32). Hemoglobin drop has been shown to correlate with PPH severity, and the most significant decline occurs within the first 24 h postdelivery (29). Second, there was a significant difference in age between the non-PPH and severe PPH groups. As fibrinolysis might be influenced by age and complications might increase in high-risk pregnancies in older women, age might have acted as a confounder in the present study. Since the size of the severe PPH group was extremely small, it was not possible to perform age-matched comparisons of the results. However, no significant correlation was found between age and TPA-test lysis time in our study population of parturient women between 20 and 45 years of age. Therefore, it seems unlikely that age differences would have exerted significant bias on our measurements. Nevertheless, age should be considered a potential confounder in future studies, especially if individuals of older age are studied. Furthermore, it must be mentioned that POC-VET fibrinolytic potential, measured by performing the ClotPro TPA-test, does not allow for statements about local fibrinolysis at the uteroplacental unit level, as it measures fibrinolytic potential in the systemic circulation. Although early administration of TXA was already recommended by international guidelines (22, 33) at the time of data collection, the recommendations were based on moderate evidence. During that time, the local standard was to administer TXA more restrictively than it is done today. Only a few patients included in the PPH group received TXA.

## 5. Conclusion

In the present study, we demonstrated that women who developed severe PPH presented with increased POC-VET fibrinolytic potential before experiencing overt bleeding, but this increased fibrinolytic potential alone was not sufficient for predicting PPH. We interpret fibrinolytic potential as a moderating variable that increases the risk of developing coagulopathy, although high fibrinolytic potential alone is not sufficient for causing excessive bleeding. Further research is needed to integrate fibrinolytic potential into established risk scores to refine their predictive ability and evaluate its use to guide targeted antifibrinolytic therapy in PPH and other fields.

## Data availability statement

The raw data supporting the conclusions of this article will be made available by the authors, without undue reservation.

## Ethics statement

The studies involving human participants were reviewed and approved by Ethics Committee of the Medical Faculty of Heidelberg, Heidelberg, Germany. The patients/participants provided their written informed consent to participate in this study.

## Author contributions

DG took responsibility for the content of the manuscript and acted as the guarantor for the article. FS, DG, HF, MF, KT, and MD were responsible for the planning and design of the study. PB was responsible for data acquisition and data integrity. TN-S, JS, MW, and HF helped recruit the patients. DG and FS were responsible for data analysis, interpretation, and wrote the manuscript with input from all other authors. HS, MAW, and SH gave substantial advice for manuscript writing and critically reviewed the manuscript. All authors have read and agreed to the published version of the manuscript.
