# Supplementary material for: Fibrinolytic potential as a risk factor for postpartum hemorrhage
Source: Front Med (Lausanne). 2023 Sep 8;10:1208103. doi: 10.3389/fmed.2023.1208103 (PMC10516290; doi:10.3389/fmed.2023.1208103)
Supplement: Supplementary file 1 [file Data_Sheet_1.docx]

FYPPREG - Supplemental

**sTable 1. Estimated blood loss and clinical outcome**

|  | **non PPH**  **n=171** | **mild PPH**  **n = 25** | **severe PPH**  **n=10** | ***p***  **non**  **vs. mild** | ***p***  **non**  **vs. severe** | ***p***  **mild vs. severe** |
| --- | --- | --- | --- | --- | --- | --- |
| **EBL [ml]** | 319 ± 83 | 718 ± 343 | 1145 ± 790 | **< .01** | **< .01** | **< .01** |
| **∆Hb [g/dl]** | 1.2 ± 0.8 | 2.3 ± 1.1 | 4.8 ± 0.9 | **< .01** | **< .01** | **< .01** |
| **HLOS [d]** | 3.6 ± 0.8 | 3.6 ± 0.7 | 4.2 ± 0.4 | 0.71 | **0.01** | 0.05 |
| **ICU admission n (%)** | 2 (1.2%) | 2 (8%) | 5 (50.0%) | 0.02 | **< .01** | **< .01** |
| **RBC Transfusion n (%)** | 0 (0.0%) | 0 (0.0%) | 3 (30.0%) | - | **< .01** | **< .01** |
| **Shock n (%)** | 0 (0.0%) | 0 (0.0%) | 3 (30.0%) | - | **< .01** | **< .01** |
| **Revision surgery n (%)** | 0 (0.0%) | 0 (0.0%) | 4 (40.0%) | - | **< .01** | **< .01** |

EBL=estimated blood loss, ∆Hb=hemoglobin drop from antepartum (T0) to first day postpartum (T2), HLOS = hospital length of stay, ICU = Intensive care unit, RBC = red blood cells. Data are presented as mean ± SD, or absolute and relative counts.

**sTable 2: Correlation TPA-test lysis time values to markers of fibrin consumption and fibrinolytic inhibitors**

| **Correlation with TPA-test lysis time** | **fibrinogen** | **d-dimers** | **alpha-2 antiplasmin** | **PAI-1** |
| --- | --- | --- | --- | --- |
| **T0** |  |  |  |  |
| **spearman correlation coefficoent** | 0.441 | -0.233 | 0.225 | 0.247 |
| **p- value** | <.0001 | 0.0012 | 0.0019 | 0.0006 |
| **T1** |  |  |  |  |
| **spearman correlation coefficoent** | 0.514 | 0.304 | 0.313 | 0.271 |
| **p- value** | <.0001 | <.0001 | <.0001 | 0.0004 |
| **T2** |  |  |  |  |
| **spearman correlation coefficoent** | 0.461 | -0.392 | 0.358 | 0.168 |
| **p- value** | <.0001 | <.0001 | <.0001 | 0.0249 |

**sTable 3: fibrinolytic potential in subgroups according to occurrence of uterine atony**

|  | **non PPH**  **n=171** | **mild PPH**  **n = 25** | **severe PPH**  **n=10** |
| --- | --- | --- | --- |
| **with uterine atony** | n = 0 | n = 2 | n = 2 |
| T0 | - | 338 ± 21 | 228 ± 32 |
| T1 | - | 262 ± 0 | 191 ± 41 |
| T2 | - | 455 ± 0 | 250 ± 0 |
| **without uterine atony** | n = 171 | n = 23 | n = 8 |
| T0 | 249 ± 41 | 244 ± 29 | 229 ± 21 |
| T1 | 235 ± 41 | 226 ± 33 | 205 ± 30 |
| T2 | 270 ± 58 | 256 ± 39 | 291 ± 97 |

TPA-test lysis time [s] in subgroups according to occurrence of uterine atony. Values are stated as mean ± standard deviation. T0=antepartum, T1=30-60min after placental separation, T2= first day postdelivery.

**sTable 4: hematocrit and hemoglobin values over the course of delivery**

|  | **non PPH**  **n=171** | **mild PPH**  **n = 25** | **severe PPH**  **n=10** | ***p***  **non**  **vs. mild** | ***p***  **non**  **vs. severe** | ***p***  **mild vs. severe** |
| --- | --- | --- | --- | --- | --- | --- |
| **Hct** |  |  |  |  |  |  |
| T0 | 0.36 ± 0.03 | 0.36 ± 0.03 | 0.37 ± 0.04 | 0.56 | 0.13 | 0.32 |
| T1 | 0.34 ± 0.03 | 0.33 ± 0.03 | 0.31 ± 0.04 | 0.64 | **0.01** | **0.04** |
| T2 | 0.32 ± 0.03 | 0.29 ± 0.03 | 0.24 ± 0.05 | **0.0002** | **<.0001** | **<.0001** |
| **Hb** |  |  |  |  |  |  |
| T0 | 12.2 ± 1 | 12.4 ± 0.9 | 12.7 ± 1.2 | 0.40 | 0.13 | 0.39 |
| T1 | 11.5 ± 1 | 11.3 ± 1 | 10.2 ± 1.5 | 0.32 | **0.0006** | **0.01** |
| T2 | 11 ± 1.1 | 10.1 ± 1.1 | 7.9 ± 1.5 | **0.0004** | **<.0001** | **<.0001** |
| **Thrombocytes** |  |  |  |  |  |  |
| T0 | 224 ± 64 | 217 ± 55 | 222 ± 64 | 0.61 | 0.93 | 0.83 |
| T1 | 206 ± 58 | 195 ± 51 | 205 ± 84 | 0.39 | 0.97 | 0.67 |
| T2 | 218 ± 62 | 203 ± 50 | 210 ± 60 | 0.25 | 0.67 | 0.77 |
| vWF activity |  |  |  |  |  |  |
| T0 | 220.2 ± 83.7 | 221.8 ± 84.8 | 275.9 ± 85.6 | 0.93 | **0.04** | 0.09 |
| T1 | 243.5 ± 88.9 | 252 ± 97.9 | 250 ± 119.3 | 0.67 | 0.84 | 0.96 |
| T2 | 259.9 ± 76.5 | 238.6 ± 77.0 | 266.3 ± 83.4 | 0.20 | 0.82 | 0.38 |
| vWF antigen |  |  |  |  |  |  |
| T0 | 249.9 ± 103.5 | 262.1 ± 92.5 | 323.1 ± 108.3 | 0.58 | **0.03** | 0.11 |
| T1 | 271.4 ± 96.6 | 285 ± 106.2 | 300 ± 125.2 | 0.53 | 0.43 | 0.71 |
| T2 | 275.1 ± 88.2 | 260.9 ± 79.6 | 279.1 ± 82.3 | 0.45 | 0.90 | 0.61 |
| XIII activity |  |  |  |  |  |  |
| T0 | 92.3 ± 20.5 | 95 ± 23.1 | 91.8 ± 24.8 | 0.56 | 0.93 | 0.68 |
| T1 | 84.5 ± 23.5 | 86.1 ± 23.3 | 72.3 ± 27.4 | 0.76 | 0.15 | 0.15 |
| T2 | 83 ± 20.7 | 75.9 ± 16.4 | 61.7 ± 17.7 | 0.10 | **0.003** | 0.09 |

**sFigure 1: D-dimer-fibrinogen ratio by group and timepoint**


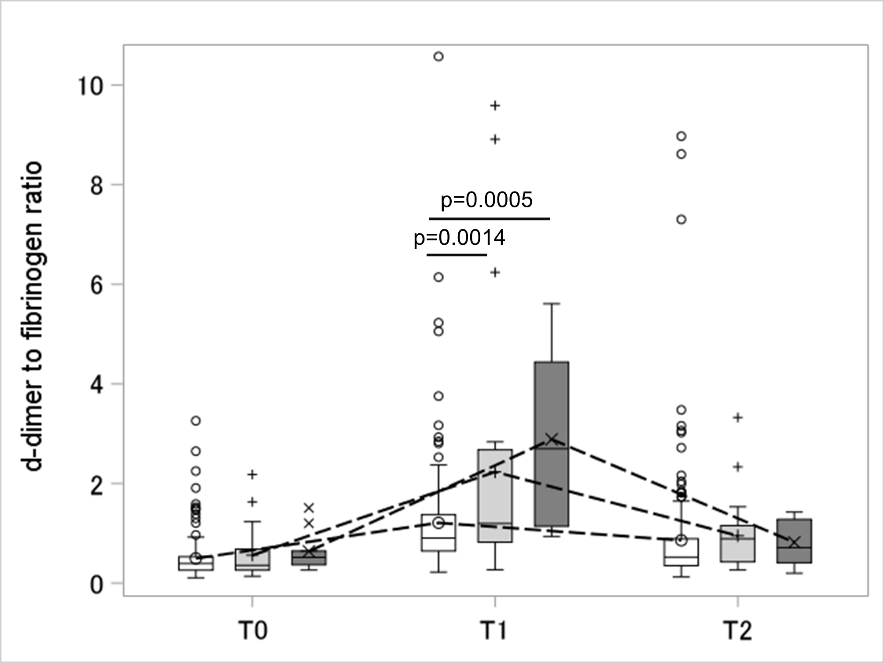


Box and whispers plot: box indicates Q1-Q3 inter-quartile range, horizontal line within the box shows median value, marker inside the box displays the mean, whiskers indicates observation nearest to the fence (=1.5 IQR), markers below and above the fences demonstrate observations above and below the fences; T0=antepartum, T1=30-60min after placental separation, T2= first day postdelivery, groups: white = non PPH, light gray= mild PPH, dark gray = severe PPH. PPH = postpartum hemorrhage,

D-dimer to fibrinogen ratios differ significantly at T1 timepoint between non PPH to mild and severe PPH respectively. No significant differences were found at hospital admission and first day postpartum.
